# Editorial: Back to the future: on the road towards precision psychiatry, volume II

**DOI:** 10.3389/fpsyt.2023.1355085

**Published:** 2024-01-08

**Authors:** Paul C. Guest, Stefan Borgwardt, Johann Steiner

**Affiliations:** ^1^Department of Psychiatry, Otto-von-Guericke-University Magdeburg, Magdeburg, Germany; ^2^Laboratory of Translational Psychiatry, Otto-von-Guericke-University Magdeburg, Magdeburg, Germany; ^3^Laboratory of Neuroproteomics, Department of Biochemistry and Tissue Biology, Institute of Biology, University of Campinas, Campinas, Brazil; ^4^Department of Psychiatry and Psychotherapy, Translational Psychiatry, Lübeck, Germany; ^5^Center for Behavioral Brain Sciences, Magdeburg, Germany; ^6^Center for Health and Medical Prevention, Magdeburg, Germany; ^7^German Center for Mental Health (DZPG), Center for Intervention and Research on Adaptive and Maladaptive Brain Circuits Underlying Mental Health (C-I-R-C), Halle-Jena-Magdeburg, Germany

**Keywords:** psychiatry, precision medicine, biomarkers, digital psychiatry, personalized medicine

In 2020, the first volume of this Research Topic was published in Frontiers in Psychiatry (1). This provided a vision of the near future with a paradigm shift toward precision psychiatry approaches that will revolutionize the way this field is investigated and breakthroughs are implemented for improved treatment of patients with mental disorders. Precision medicine is a new approach in which prevention, diagnosis and treatment takes into consideration individual variations in gene, proteome, metabolome, environmental and lifestyle factors.

However, in the last months of 2019, a new virus was detected in Wuhan China that would wreak havoc around the world for more than 2 years. This was SARS-CoV-2 which eventually infected more than 57% of people worldwide at least once, as of April 22, 2022 (2). In addition to dire effects on individual health and disruptions to society and the healthcare services, the virus had a devastating effect on mental health conditions with increases seen in depression, anxiety and post-traumatic stress disorder (PTSD). This was compounded by the fact that fewer individuals suffering from these disorders attempted to seek help from the healthcare services due to government restrictions or because the affected individuals did not think their problem was serious enough or they had fears about contracting the virus (3).

The reduced access of face-to-face mental healthcare during periods of disaster such as the pandemic called for novel solutions for continuity of patient management. Guest et al. describe the increased use of remote digital tools to promote and maintain efficacious therapeutics for individuals with psychiatric disorders. These platforms have been used as effective diagnostics, patient monitoring and treatment alternatives for people in all walks of life and professionals, including frontline healthcare workers who experienced repeated traumas during the pandemic. Digital approaches such as artificial intelligence-guided chatbots, activity-based apps and computer gameplay can be effective in improving symptoms like depression, post-traumatic stress and anxiety. They concluded that it is important to continue investing in these technologies to drive forward their integration into clinical practice. This will be especially useful during times when access to the standard facilities are restricted as in disaster scenarios. Ironically, this is a time when they will most likely be of the greatest need.

One of the fundamental challenges in neuroscience is to understand how brain activity patterns relate to behavior. Levine and Schwarzbach describe how the multivariate technique of representational similarity analysis (RSA) can be used in combination with functional magnetic resonance imaging (fMRI) to detect these patterns. This method has improved our understanding of the mechanisms involved in information encoding in local activity patterns and how these differ in individuals with and without psychiatric disorders, such as PTSD (4). The authors concluded that leveraging individualized behavioral patterns and task-related characteristics will lead to improvements in neuroimaging studies of mental depictions. This should allow analyses of differences in mental representations between individuals and to test hypotheses related to mental illnesses. It may also enable differential diagnostics and provide prognostic information as to whether or not a particular treatment will be successful.

Klein et al. attempted to unravel the controversies regarding the role of inflammation in schizophrenia. There has been conflicting evidence regarding the stimulation of both pro-inflammatory and anti-inflammatory pathways in these conditions as well as discrepancies in the findings of inflammation and immune response patterns between the central nervous system (CNS) and periphery. In addition, the mechanisms driving the entry of peripheral immune system components from the periphery into the CNS have remained elusive. They described how this process involves perivascular macrophages and dendritic cells retained in the parenchyma by the local inflammation status and this can be regulated by the presence of viral infections, which can disrupt the process of antigen presentation with persistent consequences. They also pointed out that some of the discrepancies across studies which have measured cerebrospinal fluid levels of low abundance cytokines may be confounded due to improper determination of the lower limits of detection (LOD) in multiplex analyses, in line with a recent study (5). They suggest that following strict protocol guides for determining the LOD will help to increase confidence in the results as would the use of more sensitive assay methods. Finally, they describe how further studies of these inflammation and immune-related pathways will increase our understanding of the pathophysiological mechanisms involved in schizophrenia and help us to identify new biomarkers and drug targets.

In line with the above, Vasilevska et al. show that biological links exist between viral infection, inflammation and mental illness. They describe the major elements involved in the biological interaction between the immune system and the brain. Most importantly, they proposed a stepwise system for the diagnosis and potential treatment of outcomes such as autoimmune-encephalitis as a benchmark guide in clinical practice and in the particular case of reactions to viral infections. This scheme is guided by clinical warning signs and allows for a fast and accurate diagnosis and commencement of immunotherapy as needed, in line with other published findings on identification of autoimmune encephalitis in psychiatric patients (6). If progressive psychiatric symptoms are detected with neurological signs, CSF analysis may be needed to identify the potential presence of antineuronal autoantibodies. If present, immunosuppression by corticosteroid and/or immunoglobulin administration can be performed, or immunoadsorption/plasmapheresis can be attempted. If these approaches fail, more specific immunotherapies can be applied using monoclonal antibodies such as rituximab. The timeliness of this procedure is critical as it would allow early intervention for improved patient outcomes.

Biomarker profiling methods such as metabolomics can allow the simultaneous analysis of hundreds of metabolites in blood at a time. Although several studies have now been performed in schizophrenia, these have produced inconsistent results and few have linked the relationship of changes metabolites with psychiatric symptoms. Okamoto et al. carried out a metabolomics analysis of serum from hospitalized patients with chronic schizophrenia compared to healthy controls. They used capillary electrophoresis Fourier transform mass spectrometry (CE-FTMS) for the analysis in combination with hierarchical cluster, principal component (PCA), logistic and linear regression and receiver operating characteristic (ROC) analyses, to analyse the differences in metabolites between the samples to identify those related to schizophrenia symptoms. That found that most metabolites such as those involved in glutamate metabolism and the urea cycle were lower in abundance in patients compared to controls which could be used to distinguish the two populations with high precision. Furthermore, the metabolites gamma-glutamyl-valine and gamma-glutamyl-phenylalanine showed significant negative correlations with positive and negative syndrome scale (PANSS) total and general scores and tetrahydrouridine was significantly positively correlated with PANSS negative scores. The findings support clinical studies aimed at developing pharmacological treatments targeting glutamate metabolism for efficacy in patients at specific stages of disease or in patient subgroups with similar metabolite profiles.

The major difficulty in prescribing exact treatment for individuals with psychotic disorders is disease heterogeneity. For this reason, diagnosis, prognosis and treatment course can vary wildly. In addition, patients can experience adverse events which can lead to poor treatment adherence and variations in individuals in drug response could occur due to factors such as age, sex, ethnicity, body composition, genetics and metabolism. Like most other drugs, antipsychotics are metabolized by the highly polymorphic cytochrome P450 (CYP450) enzymes in the liver. Marcos-Vadillo et al. describe a pharmacogenomic approach in guiding the treatment of a psychotic patient who presented adverse events, which appeared to be caused by his genetic profile and drug interactions. This patient was profiled as having a novel CYP2D6 allele paired with a non-functional allele which decreased the activity of this CYP450 enzyme and may have contributed to the adverse events after treatment with specific antipsychotics. Guided by his pharmacogenomics profile, the patient was administered a different antipsychotic with a final report of no adverse events. The authors conclude by suggesting that application of this methodology could help to reduce pharmaceutical exacerbations, hospitalizations and economical costs associated with adverse drug events in a personalized medicine manner.

The technique of retinal electrophysiology has also been used to study neural functioning and to identify biomarkers for enhanced diagnosis, prognosis and prediction of treatment response in patients with psychiatric disorders (7). Schwitzer et al. describe the use of electroretinography (ERG) to identify implicit time or peak time dysfunctions in the retinas of patients. These findings suggest changes in neuronal processing speed in the retinas which can be linked with the same changes in neurons in the brain. They suggest the combined use of synchronized retinal and cortical electrophysiology using ERG and visual evoked potentials (VEPs) to firmly establish this link. They present evidence at the anatomical, physiological and methodological levels to support this. They conclude that the an integrated retinocortical time (RCT) parameter may have utility in psychiatric research, especially in combination with signal processing and machine learning tools to establish the most robust biomarker features for use in precision psychiatry.

At the time of publication of this Research Topic, psychiatric research is still recovering in the aftermath of the SARS-CoV-2 pandemic which has had unprecedented effects on mental health around the world. This disastrous situation has also brought to light the importance of precision psychiatry though the increased need for biomarker readouts reflecting brain function and immune status, metabolomics and pharmacogenomics to guide treatment options, and digital approaches to help maintain treatment standards in disaster scenarios such as those caused by the pandemic. It is hoped that this move toward precision psychiatry will continue to evolve over the coming years toward improved prediction, diagnosis, prognosis and treatment guidance for individuals suffering with mental disorders.

## Author contributions

PG: Conceptualization, Writing—original draft, Writing—review & editing. SB: Conceptualization, Writing—original draft, Writing—review & editing. JS: Conceptualization, Writing—original draft, Writing—review & editing.
